# Inhibitory Activity of Silver Nanoparticles Synthesized Using *Lycopersicon Esculentum* against Biofilm Formation in *Candida* Species

**DOI:** 10.3390/nano9111512

**Published:** 2019-10-23

**Authors:** Jeong Su Choi, Ji Woong Lee, Un Chul Shin, Min Woo Lee, Dae Jin Kim, Suhng Wook Kim

**Affiliations:** 1 Department of Health and Safety Convergence Science, Graduate School, Korea University, 145 Anam-ro, Seongbuk-gu, Seoul 02841, Korea; guesschjs@korea.ac.kr (J.S.C.); mozorida78@korea.ac.kr (J.W.L.); 2 Institute of Health Science, College of Health Science, Korea University, 145 Anam-ro, Seongbuk-gu, Seoul 02841, Korea; fdlnlmt@korea.ac.kr (U.C.S.); leemw@korea.ac.kr (M.W.L.); 3 Department of Neurology, Pusan National Yangsan Hospital, Yangsan 50612, Korea; qkqheowls@hanmail.net

**Keywords:** *Lycopersicon esculentum*, silver nanoparticles, *Candida*, biofilm

## Abstract

This paper investigated the antifungal and antibiofilm activity of silver nanoparticles synthesized with *Lycopersicon esculentum* extracts against *Candida* species. *Lycopersicon esculentum* extracts obtained by homogenization were mixed with silver nitrate to synthesize silver nanoparticles. Analysis of the particle characteristics by UV–Vis spectrophotometry, scanning electron microscopy (SEM), energy dispersive X-ray spectroscopy (EDAX), dynamic light scattering (DLS), and Fourier transform infrared spectroscopy (FT-IR) confirmed that the *Lycopersicon esculentum* extracts effectively served as reductants and capping agents. Minimum inhibitory concentration (MIC) tests were conducted to confirm antifungal activity against *Candida* species. In all the tested species, the silver nanoparticles inhibited the growth of *Candida*. Moreover, the SEM images of *Candida* species treated with silver nanoparticles synthesized using natural extracts of *Lycopersicon esculentum* showed that silver nanoparticles adhered to the surface of *Candida,* which induced pore formation in the membranes and prevented their normal growth. Ultimately, these abnormal forms of *Candida* were thought to be less able to form biofilms than normal *Candida*. The antifungal and antibiofilm activities of silver nanoparticles against *Candida* are expected to be utilized in various fields and contribute in particular to developments in nanomedicine.

## 1. Introduction

The synthesis of nanoparticles has been studied for some time, and various attempts have been made to apply them to advance the field of nanomedicine [1]. Nanoparticles have diverse features that are dependent on the metallic type, size, and shape of the particles. Specifically, silver nanoparticles are well known for their biocidal properties, which include antibacterial, antifungal, antiviral, and anticancer activities [2]. For this reason, silver nanoparticles take center stage as potential therapeutic biomolecules. Additionally, as the tendency toward consideration of economic and environmental benefits becomes more pronounced, efforts to find efficient synthesis methods that do not involve hazardous and pollutive chemical agents have grown [3]. Green synthesis utilizes components originating from natural resources such as vitamins, sugars, plant extracts, biodegradable polymers, and microorganisms, which act as reductants and capping agents [4]. The most common silver nanoparticles synthesis method is a chemical synthesis method using sodium borohydride (NaBH_4_). Sodium borohydride reduces Ag^+^ ions to Ag^0^ in aqueous silver nitrate solution [5].

However, this method is unsafe because it requires the handling of harmful reagents during the synthesis process, and the products have undesirable impacts on the environment. Moreover, the silver nanoparticles produced by chemical synthesis are highly toxic and have low biocompatibility, making them unsuitable for use in nanomedicine applications [6,7]. However, the green synthesis method has characteristics that overcome the disadvantages of chemical synthesis, including: (1) a simple procedure; (2) suitability for large-scale applications; (3) high colloidal stability; (4) outstanding biocompatibility; (5) cost-effectiveness; and (6) eco-friendliness [8]. Plant extracts could be ideal candidates for green synthesis. Many vigorous attempts have been made to select candidate plant extracts for green synthesis, and silver nanoparticles have already been synthesized in various sizes and shapes using plant extracts [9]. Table 1 shows the various biological plant sources used in green synthesis. Plant extracts contain polysaccharides and phytochemicals. Hydroxyl groups and hemiacetal reducing ends in the polysaccharides play significant roles in nanoparticle synthesis as reductants and capping agents. Phytochemicals such as flavonoids, terpenoids, and polyphenols contribute to the reduction of Ag^+^ and the stability of Ag^0^ [10]. In this study, *Lycopersicon esculentum* (red tomato) extracts were used to synthesize silver nanoparticles. *Lycopersicon esculentum* contains several components, including vitamins A and C, ß-carotene, lycopene, organic acids, and phenolic compounds, which are expected to act as reductants and capping agents [11]. In this study, we examined the properties of silver nanoparticles synthesized by *Lycopersicon esculentum* extracts and their fungicidal activity against *Candida* species. The results suggest the potential of silver nanoparticles as therapeutic agents.

## 2. Materials and Methods

### 2.1. Preparation of Fruit Extracts

Fresh *Lycopersicon esculentum* were obtained from the local market (Seoul, Korea). The *Lycopersicon esculentum* surface was rinsed well using deionized water. To obtain the fruit flesh, the skin was peeled off and the fruit was chopped into small pieces. Then, the *Lycopersicon esculentum* juice was extracted by homogenization. The juice was centrifuged at 8000 rpm for 15 min to remove the fruit debris, and the obtained supernatants were filtered using Whatman No.1 filter paper. The aqueous filtered extracts were filtered once more through a 0.25-μm syringe filter to remove remaining contaminants. The filtrates were stored at 4 °C until synthesis of the nanoparticles.

### 2.2. Preparation of Silver Nanoparticles

Silver nitrate (AgNO_3_) was purchased from Sigma-Aldrich (St Louis, MO, USA) for the green synthesis of silver nanoparticles. *Lycopersicon esculentum* extract (10 mL) was dropped into 40 mL of 5 mM silver nitrate solution and incubated at room temperature under mild agitation. The control AgNO_3_ solution was made without the addition of *Lycopersicon esculentum* extracts. To determine the silver nanoparticles (AgNPs) concentration, the mixture of silver nitrate and *Lycopersicon Esculentum* extracts containing the silver nanoparticles was centrifuged at 13,000 rpm for 15 min. The AgNPs pellet was washed with deionized water and the washing process was repeated three times to remove any biological molecules. Finally, freeze-drying was conducted to obtain the purified AgNPs powder, and this powder was dissolved in deionized water to make a stock solution.

### 2.3. Characterization of the Silver Nanoparticles

UV–Vis spectrophotometry is one of the most basic analyses used to confirm the synthesis of nanoparticles. To study the time-based yield of particle synthesis, the *Lycopersicon esculentum* extract and silver nitrate mixture were measured over an absorbance range of 300 to 800 nm every three min until no more absorbance changes were detected. Scanning electron microscopy (SEM) images provide specialized information on morphological properties, such as size and shape. The colloidal silver nanoparticle solution was smeared on cover glass and allowed to dry overnight. Then, the cover glass was placed on a copper stub and bound to carbon tape. The SEM images were obtained with a JEOL, Ltd. (Tokyo, Japan) instrument (JSM-6701F) at 10 kV accelerating voltage and energy dispersive X-ray spectroscopy (EDAX) analysis was performed simultaneously with instrumentation contained in the SEM to confirm the presence of elemental silver. The particle size distribution was measured using dynamic light scattering (DLS, Zetasizer nano S90 system, Malvern, UK) at 25 °C. Fourier transform infrared spectroscopy (FT-IR) was used to investigate the functional groups of the biomolecules that contributed to the synthesis of the silver nanoparticles. FT-IR (Spectrum 100, Perkin Elmer Corporation, Norwalk, CT, USA) measurements were made between 4000 and 400 cm^−1^.

### 2.4. Antifungal and Antibiofilm Activity of the Silver Nanoparticles

#### 2.4.1. Minimum Inhibitory Concentration of Silver Nanoparticles against *Candida* Species

The minimum inhibitory concentration (MIC) for planktonic *Candida* species such as *C. albicans* (ATCC 90028), *C. parapsilosis* (ATCC 90018), and *C. glabrata* (ATCC 90030) was determined with minor modifications of the broth microdilution method described by the Clinical and Laboratory Standards Institute [27]. In brief, serial two-fold dilutions of silver nanoparticles were prepared in final concentrations ranging from 2 to 64 µg/mL and added to 96-well flat-bottom microtiter plates. Suspensions of *Candida* species adjusted to 0.5 McFarland (1–5 × 10^6^ CFU/mL) were suspended in RPMI 1640 medium at a final density of 0.5–2.5 × 10^3^ CFU/mL and added to test wells containing the diluted silver nanoparticles. All plates were incubated at 37 °C for 48 h without agitation. After incubation, the cells were resuspended in each well by pipetting in order to remove the effect of precipitated cells on absorbance. The absorbance at 620 nm was measured using a microplate reader (SpectraMax 190, Molecular Devices, Downingtown, PA, USA) to confirm the growth of fungi. The lowest concentration at which the absorbance decreased below 50% compared to the control was considered the MIC. 

#### 2.4.2. Inhibitory Effect of Silver Nanoparticles on Pre-Formed Biofilms

To confirm the inhibitory effect of silver nanoparticles on pre-existing biofilm, *Candida* biofilms were mounted on the 96-well plate surface for 24 h at 37 °C as described above, without silver nanoparticle treatment. After washing with PBS to remove floating cells, the selected wells were filled with fresh RPMI 1640 medium and treated with silver nanoparticles at the same concentrations as those used in the MIC test. The plates were then incubated for another 24 h at 37 °C. After incubation, the biofilms attached to the bottom of the plate were suspended by pipetting and the absorbance was measured at 620 nm using a microplate reader. The concentration of silver nanoparticles causing a turbidity decrease below 50% was considered the MIC for biofilm formation.

#### 2.4.3. Morphology of *Candida* Biofilm Treated with Silver Nanoparticles 

Scanning electron microscopy images were obtained to confirm the inhibitory effect of silver nanoparticles on biofilm formation. Suspensions of *Candida* species were adjusted to 0.5 McFarland in RPMI 1640 medium and inoculated into six-well plates containing custom-made 20 mm × 20 mm polystyrene coverslips. The polystyrene coverslips were preincubated at 37 °C for 2 h to allow attachment of the *Candida* species. After preincubation, the coverslips were gently washed with PBS to remove only non-adherent cells. The test wells were then filled with medium containing 64 µg/mL silver nanoparticles and incubated to develop biofilms at 37 °C for 48 h. The control wells were refilled with fresh RPMI 1640 medium only. Following formation of the biofilms, the biofilms on the polystyrene coverslips were washed with PBS and fixed overnight in 2.5% glutaraldehyde in PBS. A graded series of ethanol (70%, 80%, 90%, and 100%) were used to dehydrate the coverslips and they were air dried overnight in a desiccator. After processing, the samples were coated with platinum using an automatic magnetron sputter coater system and were observed by scanning electron microscopy. Representative images of the effect of silver nanoparticles on the entire sample surface are presented.

### 2.5. Statistical Analysis

MIC tests were conducted in triplicate. The results were analyzed by one-way ANOVA, and Tukey post-tests were used to compare multiple groups using the SPSS software package (SPSS, Version 12.0, Chicago, IL, USA). All data in the figures are expressed as the mean and standard deviation of three independent experiments and statistical significance was accepted for *p*-values less than 0.05.

## 3. Results and Discussion

### 3.1. Characterization of the Silver Nanoparticles (AgNPs)

#### 3.1.1. UV–Vis Spectral Analysis

**Figure 1:**
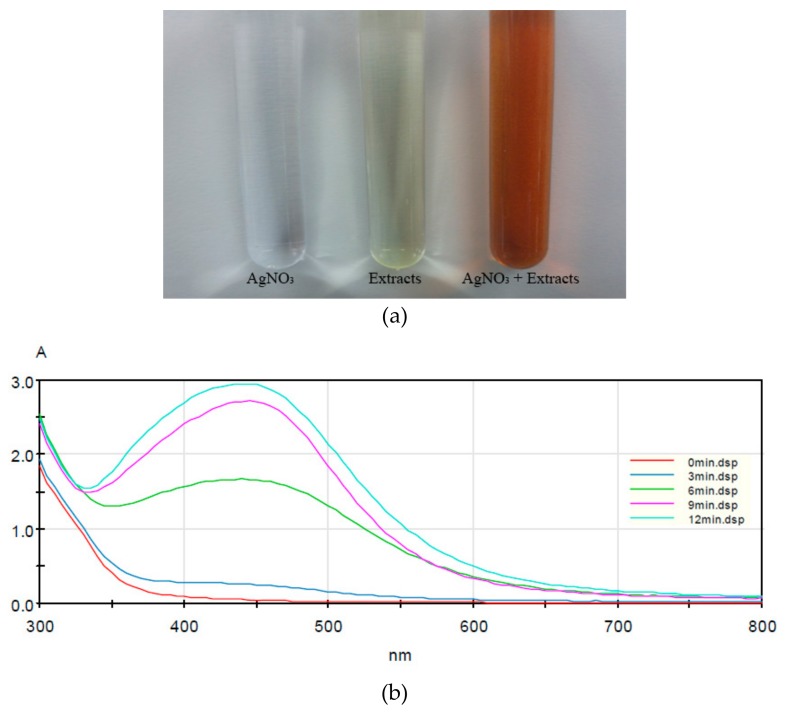
Synthesis of silver nanoparticles: (**a**) color of the silver nitrate solution, the *Lycopersicon esculentum* extract, and the mixture; and (**b**) change in UV–Vis spectrum due to silver nanoparticle synthesis.

Color change indicated the synthesis of AgNPs occurring by the reduction of Ag+ ions. The silver nitrate and *Lycopersicon esculentum* extract mixture gradually changed from colorless to yellow, then to reddish-brown after about 10 min, suggesting the synthesis of silver nanoparticles. The control AgNO_3_ solution, on the other hand, remained colorless (Figure 1a). To confirm the synthesis of AgNPs, the reaction mixture was analyzed by UV–Vis spectrophotometry. We observed a specific pattern emerging—a peak referred to as surface plasmon resonance (SPR) [28]. The SPR pattern is dependent upon characteristics of the individual metal particles, such as size and shape, as well as the dielectric properties of the medium used for synthesis and the inter-nanoparticle coupling interactions [29]. In this study, the λ_max_ was observed at 445 nm (Figure 1b). The intensity of the SPR band increased with reaction time, indicating synthesis of the AgNPs. The maximum absorbance was recorded at 12 min and indicated the end of the reaction.

#### 3.1.2. Scanning Electron Microscopy (SEM) Image

**Figure 2:**
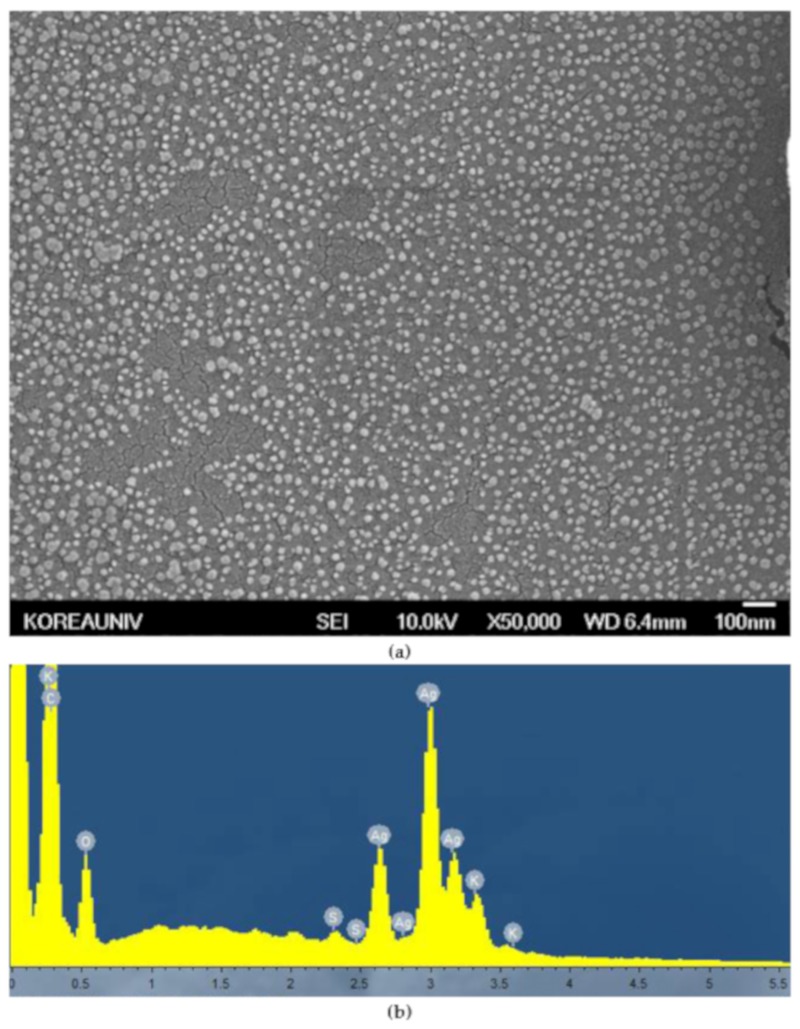
(**a**) Scanning electron microscopy (SEM) image and (**b**) energy dispersive X-ray spectroscopy (EDAX) spectrum of the synthesized silver nanoparticles (AgNPs). Silver nanoparticles were coated with platinum before analysis. The magnification level is 50,000×.

Figure 2a shows an SEM image of AgNPs synthesized from *Lycopersicon esculentum* extract. SEM analysis showed that the shape of the AgNPs was predominantly spherical and the particle sizes ranged from 10 to 50 nm, without any larger aggregations. This result indicated that the *Lycopersicon esculentum* extracts worked effectively as a reductant and capping agent to synthesize and disperse the AgNPs. Figure 2b shows strong signals of silver atoms on the EDAX spectrum, confirming the synthesis of AgNPs, and other signals for carbon, oxygen, sulfur, and potassium, indicating the presence of organic substances in the *Lycopersicon esculentum* extracts.

#### 3.1.3. Dynamic Light Scattering (DLS)

**Figure 3:**
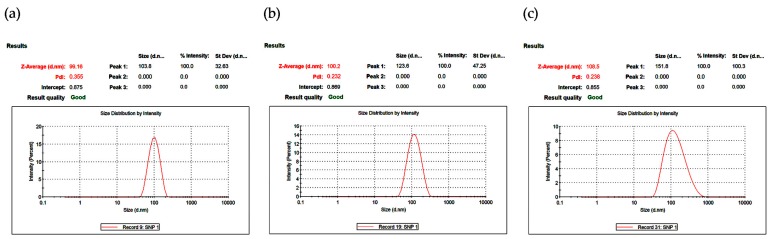
Particle size distribution curve of synthesized AgNPs at: (**a**) 24 h; (**b**) 48 h; and (**c**) 72 h.

DLS was used to analyze the size distribution and colloidal stability of AgNPs for 72 h (Figure 3). The z-average values were measured as 99.16 nm at 24 h (Figure 3a), 100.2 nm at 48 h (Figure 3b), and 108.5 nm at 72 h (Figure 3c), indicating that the particle size remained nearly constant for 48 h. In addition, narrow particle size distributions were observed by the low polydispersity index (PDI) of 0.232 at 48 h (Figure 3b).

#### 3.1.4. Fourier Transform Infrared Spectroscopy (FT-IR)

**Figure 4:**
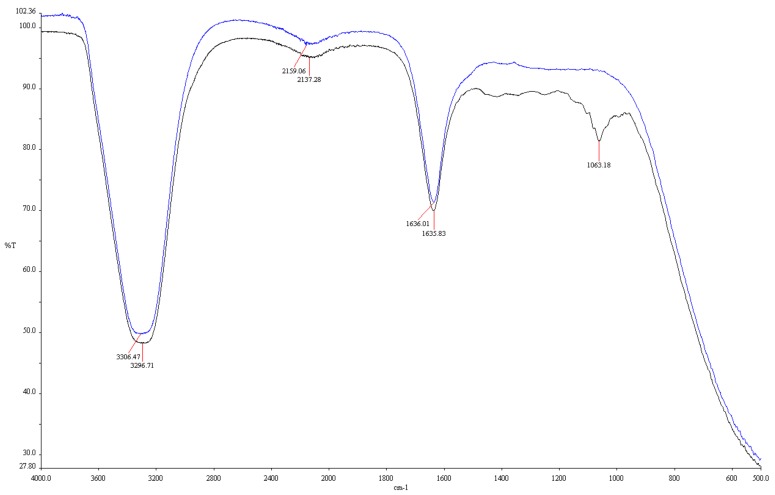
Fourier transform infrared spectroscopy (FT-IR) spectra of *Lycopersicon esculentum* extract (blue line) and biosynthesized AgNPs (black line).

FT-IR analysis showed the functional groups in the fruit extract involved in the synthesis of AgNPs. The main factors in the conversion of Ag^+^ ions to Ag^0^ are shown in the FT-IR spectra. Figure 4 shows the different patterns of AgNPs solution with *Lycopersicon esculentum* extract and the *Lycopersicon esculentum* extract alone. Distinct absorption peaks of *Lycopersicon esculentum* extract were observed at 3296.71, 2137.28, 1635.83, and 1063.18 cm^−1^. The peak at 3296.71 cm^−1^ corresponded to the stretching vibration of −OH bonds in alcohols and phenols [30]. The weak intensity peak at 2137.28 cm^−1^ was due to the stretching vibration of −C≡C− bonds in alkynyl groups [21]. The peaks at 1635.83 cm^−1^ were influenced by C=O stretching vibrations associated with flavonoids and terpenoids [31]. The peak at 1063.18 cm^−1^ was assumed to represent C–O stretching vibration in ester and ether [13]. −OH and C=O bonds were related to the reduction of Ag^+^ to Ag^0^. The functional groups contained in alcohols, phenols, and flavonoids acted as capping agents involved in the reduction and stabilization of the AgNPs [32].

### 3.2. Antifungal and Antibiofilm Activity of the Silver Nanoparticles

#### 3.2.1. Minimum Inhibitory Concentration of the Silver Nanoparticles

**Figure 5:**
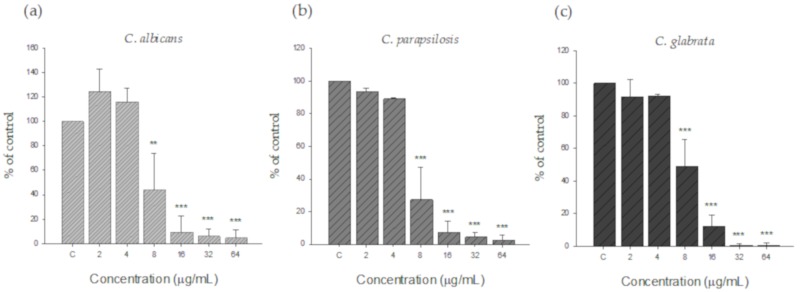
Antifungal activity of AgNPs against (**a**) *C. albicans*, (**b**) *C. parapsilosis*, and (**c**) *C. glabrata*. All species were inoculated at a final density of 0.5–2.5 × 10^3^ CFU/mL in RPMI 1640 medium. The silver nanoparticles were prepared at final concentrations ranging from 2 to 64 µg/mL.

The potential of silver nanoparticles as antifungal agents is well known. The antifungal activity of AgNPs against planktonic *C. albicans, C. parapsilosis,* and *C. glabrata* was confirmed by MIC testing. The MIC of silver nanoparticles of all *Candida* species tested was 8 µg/mL (Figure 5). No inhibition was observed in the low concentration range (2 to 4 µg/mL), but growth was significantly inhibited at 8 µg/mL or more compared to the control.

#### 3.2.2. Inhibitory Effect of Silver Nanoparticles on Pre-Formed Biofilms

**Figure 6:**
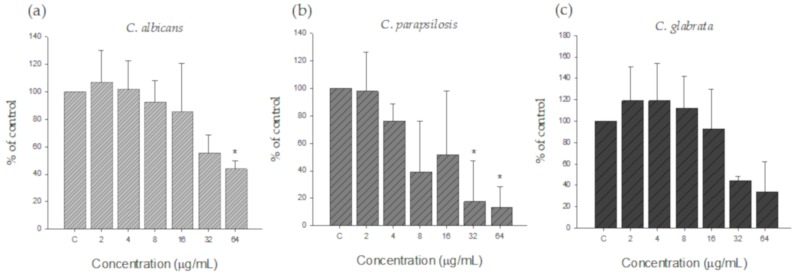
Activity of AgNPs against pre-formed biofilm of (**a**) *C. albicans*, (**b**) *C. parapsilosis*, and (**c**) *C. glabrata*. All species formed biofilm prior to treatment with AgNPs. The silver nanoparticles were prepared at final concentrations ranging from 2 to 64 µg/mL.

To confirm the inhibitory effect of silver nanoparticles on biofilm, pre-formed biofilms were treated with various concentrations of silver nanoparticles. As shown in Figure 6, silver nanoparticles showed biofilm inhibitory activity against all *Candida* species tested. However, the MIC for biofilm was higher than the MIC for planktonic cells. The MIC value for *C. albicans* and *C. glabrata* was 32 µg/mL, while the MIC for *C. parapsilosis* was 8 µg/mL, demonstrating that *C. parapsilosis* had the highest susceptibility to AgNPs and *C. glabrata* had the highest resistance to AgNPs among the three *Candida* species tested. 

#### 3.2.3. Morphology of *Candida* Biofilm Treated with Silver Nanoparticles 

**Figure 7:**
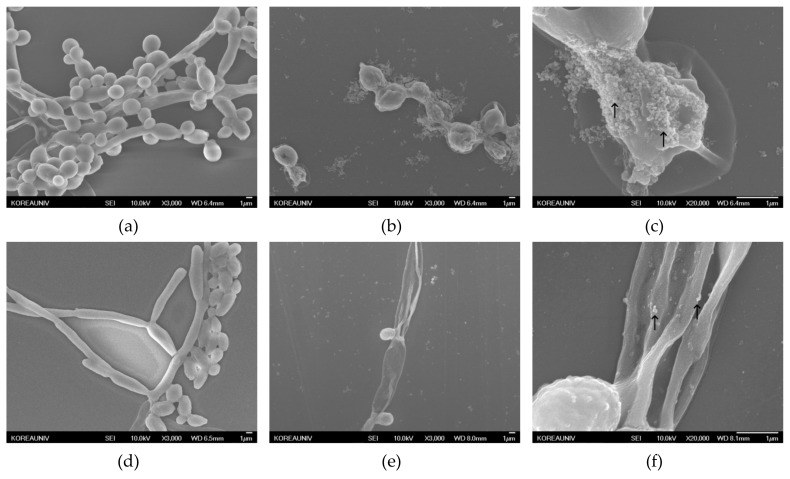

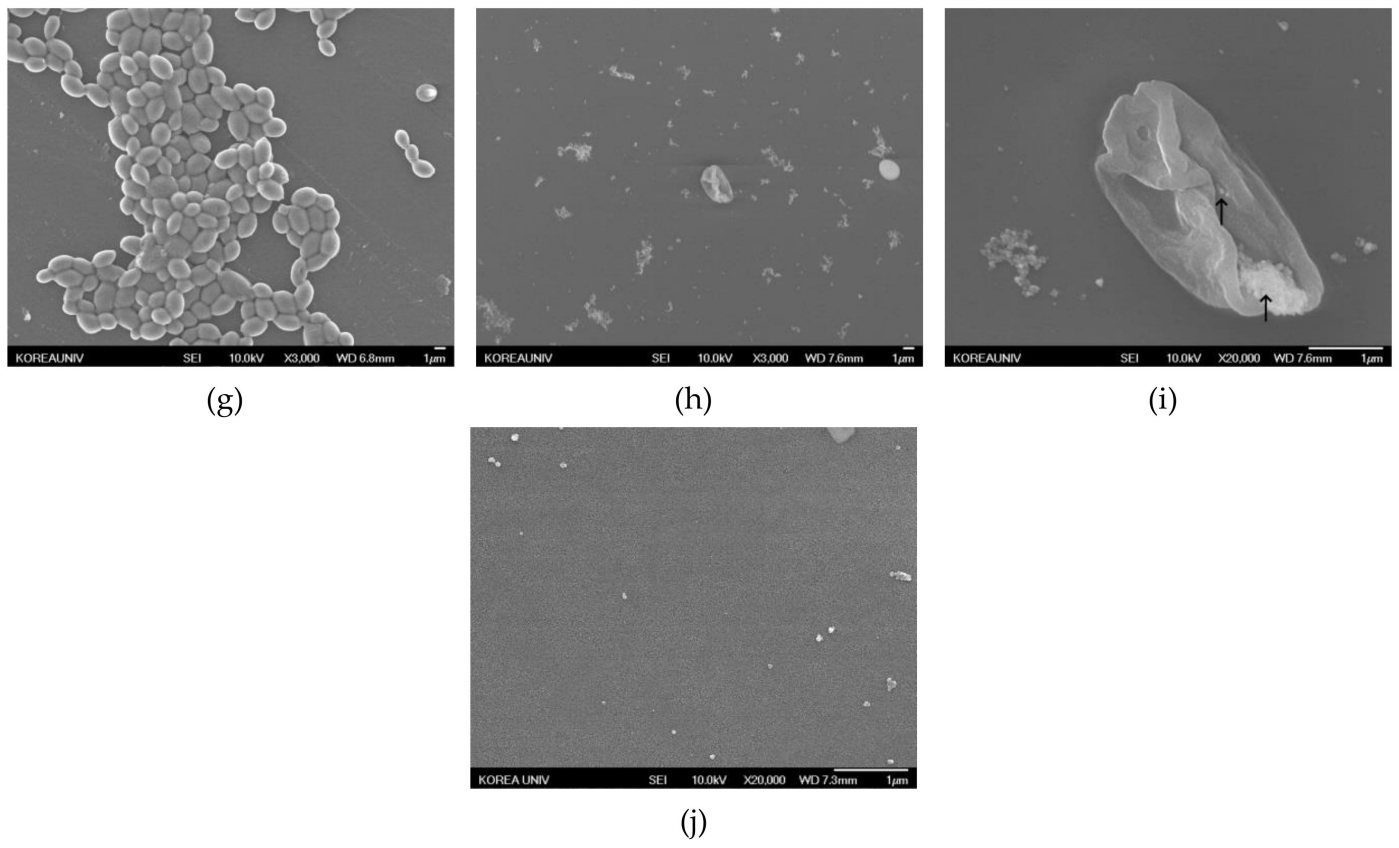
SEM images of *Candida* species treated with silver nanoparticles: (**a**) untreated *C. albicans* (3000× magnification); (**b**) *C. albicans* treated with 64 µg/mL silver nanoparticles (3000× magnification); (**c**) *C. albicans* treated with 64 µg/mL silver nanoparticles (20,000× magnification); (**d**) untreated *C. parapsilosis* (3000× magnification); (**e**) *C. parapsilosis* treated with 64 µg/mL silver nanoparticles (3000× magnification); (**f**) *C. parapsilosis* treated with 64 µg/mL silver nanoparticles (20,000× magnification); (**g**) untreated *C. albicans* (3000× magnification); (**h**) *C. glabrata* treated with 64 µg/mL silver nanoparticles (3000× magnification); (**i**) *C. glabrata* treated with 64 µg/mL silver nanoparticles (20,000× magnification, AgNPs are shown as black arrows); and (**j**) control image of AgNPs (20,000× magnification).

The antibiofilm activity of AgNPs was also verified by SEM analysis. Figure 7 shows an SEM micrograph of *Candida* species before and after treatment with AgNPs for 48 h. The biofilm formed in microbial communities by the irreversible attachment of fungal cells to substratum, interfaces, or each other is a major contributor to *Candida* pathogenicity. This physiological structure provides *Candida* species with an inherent resistance to antimicrobial agents [33,34]. *Candida* species are known to have three main cellular morphologies: yeast, pseudohyphae, and hyphae [35]. *C. glabrata* does not exhibit a heterogeneous morphology, growing only in the yeast form, and *C. parapsilosis* only forms yeasts and pseudohyphae, often referred to as giant cells. In contrast, *C. albicans* is capable of forming yeast, pseudohyphae, and hyphae. The hyphae, a germ tube feature, has clinical significance in the diagnosis of *C. albicans* [36]. The *C. albicans* control (Figure 7a) showed typical and smooth morphology, and the yeast and hyphae forms were found in the biofilm. Hyphae were not visible in *C. albicans* treated with AgNPs (Figure 7b), and only single or budding yeast with rimose membranes were seen. High-magnification images of *C. albicans* treated with AgNPs (Figure 7c) showed AgNPs attached and agglomerated to the surface of nearby yeasts (black arrows on the image). In *C. parapsilosis* (Figure 7d–f), the control biofilms (Figure 7d) were mainly composed of yeasts and pseudohyphae with regular morphology. After treatment with AgNPs (Figure 7e), yeasts with rugged membranes but no pseudohyphae were present throughout the sample. The 20,000× magnified image (Figure 7f) confirmed that the AgNPs were located on the surface of wrinkled, abnormal yeasts and pseudohyphae (black arrows on the image). In *C. glabrata* (Figure 7g–i), the biofilms of untreated *C. glabrata* (Figure 7g) were comprised only of spherical or oval-shaped blastoconidia, which were considerably smaller than *C. albicans* and *C. parapsilosis*. After *C. glabrata* was treated with AgNPs (Figure 7h), abnormal morphology, some pores, and distorted membranes appeared in the yeasts. Figure 7i (magnification at 20,000×) shows AgNPs stuck around the perforated membranes of *C. glabrata* (black arrows on the image), which were also observed with *C. albicans* and *C. parapsilosis*. The effects of AgNPs on fungal cell walls were previously reported. Kim et al. [37] described the ability of AgNPs to disrupt the membrane potential and affect the membrane dynamics in *C. albicans.* The interaction between AgNPs and the membrane structures was confirmed by TEM analysis, which indicated the formation of pits on the membrane and the formation of additional pores, which lead to cell death. The SEM image analysis in this study yielded similar results. The AgNPs were thought to cause abnormalities in the cell membranes by binding to the surface, inhibiting normal growth, and consequently reducing biofilm formation.

## 4. Conclusions

In this study, we reported that *Lycopersicon esculentum* extract played a role in the synthesis of spherical nano-sized silver particles, acting as a reductant and capping agent. UV–Vis spectral analysis confirmed the synthesis of silver nanoparticles. SEM analysis showed that the synthesized particles had nano-scale sizes, and EDAX spectrum divulged the presence of silver atoms. The functional groups involved in the bioreduction of Ag ions to AgNPs were analyzed in FT-IR spectra. Outstanding antifungal activity of the silver nanoparticles was confirmed by MIC tests against *Candida* species and SEM microscopy supported the inhibitory effect of silver nanoparticles on biofilm formation. These silver nanoparticles produced from *Lycopersicon esculentum* extract without any toxic chemical reagents have many advantages, including simple production, cost-effectiveness, eco-friendly fabrication and product, and biocompatibility. These properties will enable AgNPs to be used in diverse areas. Specifically, the application of AgNPs to fungicidal therapy may make a considerable contribution to nanomedicine.

## Figures and Tables

**Table 1:** Various plant extracts used for the green synthesis of silver nanoparticles.

No.	Plants	Part Used	Particle Size (nm)	References
1.	*Acalypha indica*	leaves	20–30	[12]
2.	*Alternanthera dentata*	leaves	50–100	[13]
3.	*Artocarpus heterophyllus*	seeds	3–25	[14]
4.	*Azadiracha indica*	leaves	50–100	[15]
5.	*Boerhaavia diffusa*	leaves	15–28	[16]
6.	*Caesalpinia coriaria*	leaves	40–50	[17]
7.	*Carica papaya*	fruits	60–80	[18]
8.	*Curcuma longa*	tubers	21–30	[19]
9.	*Delphinium denudatum*	roots	~85	[20]
10.	*Erythrina indica*	roots	20–118	[21]
11.	*Gossypium hirsutum*	leaves	10–40	[22]
12.	*Jatropha curcas*	petals	15–25	[23]
13.	*Rosa damascena*	seeds	15–27	[24]
14.	*Sambucus nigra*	fruits	20–80	[25]
15.	*Tribulus terrestris*	fruits	10–30	[26]
